# 

**Published:** 1830-11

**Authors:** 


### MONTHLY LIST OF MEDICAL BOOKS.

An Introduction to the Natural System of Botany: or, a Systematic View of the Organization, Natural Affinities, and Geographical Distribution of the whole Vegetable Kingdom; together with the uses of the most important Species in Medicine, the Arts, and Rural or Domestic Economy. By JOHN LINDLEY, F.R.S. L.S. G.S. &c. and Professor of Botany in the University of London. 8vo. pp. 374. London: Longman, 1830.

LOUDON'S HORTUS BRITANNICUS. A Catalogue of all the Plants indigenous, cultivated in, or introduced to Britain. Part I. The Linnean Arrangement, in which nearly 30,000 Species are enumerated; with the Systematic Name and Authority, Accentuation, Derivation of Generic Names, literal English and Specific Names, Synonymes, systematic and English, of both Genera and Species; Habit, Habitation in the Garden, indigenous Habitation, popular Character, Height, Time of Flowering, Colour of the Flower, Mode of Propagation, Soil, Native Country, Year of Introduction, and Reference to Figures: preceded by an Introduction to the Linnean System. Part II. The Jussieuean Arrangement, or nearly 40,000 Genera, with an Introduction to the Natural System, and a General Description and History of each Order. Edited by J. C. LOUDON, F. L., H., G., and Z. S. 8vo. pp. 576. London: Longman, 1830.

A Practical Treatise on Diseases of the Eye. By WILLIAM MACKENZIE, Lecturer on the Eye in the University of Glasgow, and one of the Surgeons to the Glasgow Eye Infirmary. 8vo. pp. 861. Longman, 1830.

A Demonstration of the Nerves of the Human Body; consisting of four Parts. Part I. the Cervical and Thoracic Portions of the Sympathetic, and the Nerves of the Thoracic Viscera. Part II. the Lumbar and Sacral Portions of the Sympathetic, and the Nerves of the Abdominal Viscera. Part III. the Cerebral Nerves. Part IV. the Spinal Nerves. By JOSEPH SWAN. Large folio. Longman, 1830.

Mr. Swan's name is a passport to the attention of the profession upon any subject connected with the anatomy or physiology of the nerves. These plates are executed in the very first style; the descriptive text is concise, yet perspicuous; the references are clear and distinct. The work will be confined to the illustration of the subjects of the two Collegial Anatomical Prizes adjudged to Mr. Swan in 1825 and 1828, by the Royal College of Surgeons. To anatomical students, and practitioners in surgery and medicine, this work will be found of great service: by its help, the former will be enabled to understand, and to store up in his memory, the lectures of his teacher upon the nervous system; and the latter, who can no longer attend the dissecting room, will very often find, by referring to it, a clue to many pathological phenomena which would be unintelligible without that kind of assistance which the ability and zeal of Mr. Swan here offers. The first part only of this very splendid and useful work is yet published.

Medico-Chirurgical Transactions, Vol. XVI. Part I. 8vo. pp. 235; two Plates. Longman, 1830.

Tronsactions the Medical and Physical Society of Calcutta. Vol. IV. 8vo. pp 449. Parberry and Allen, 1829.

Observations on Asthma, Consumption, and other Disorders of the Lungs; with Cases showing the Benefit to be derived from a Nutrient and Invigorating System of Diet and Regimen, in Pulmonary Diseases and disordered Breathing. By A. RENNIE, Surgeon, &c. Second Edition. 8vo. pp. 95. Burgess and Hill, 1830.

A Practical Treatise on General or Partial Debility, either Original or Hereditary, or from Age, Dissipation, Residence in a Tropical Climate, &c.: and on the most effectual Means of Preventing and Curing Organic Diseases, &c. which undermine the Constitution, and lay the Foundation of the Climacteric Malady, or Breaking-up of the Constitution, in both Sexes, by Diet, Exercise, and the Round-leaf Cornel, where a tonic Remedy is necessary. To which is added, an Account of the Lobelia Inflata, the celebrated American Specific for Asthma; with Remarks on the Seat, Causes, Dietetic and Medical Treatment of the different Varieties of Asthma. By S. H. ROBINSON, M.D. and others. 8vo. pp. 86. Highley, London, 1829.

Elements of Surgery. By ROBERT LISTON, Fellow of the Royal Colleges of Surgeons in London and Edinburgh; Surgeon to the Royal Infirmary, &c. Part I. 8vo. pp. 318. London: Longman and Co. 1831.

*Communications received from* Dr. and Mr. D. GRIFFIN, Dr. BARRY, *&c.*

